# The State of SLIT

**DOI:** 10.1097/WOX.0b013e3181954066

**Published:** 2009-01-15

**Authors:** Johannes Ring, Lanny Rosenwasser

**Affiliations:** 1 Executive Editor; 2 Editor-in-Chief

## 

**Johannes Ring:**
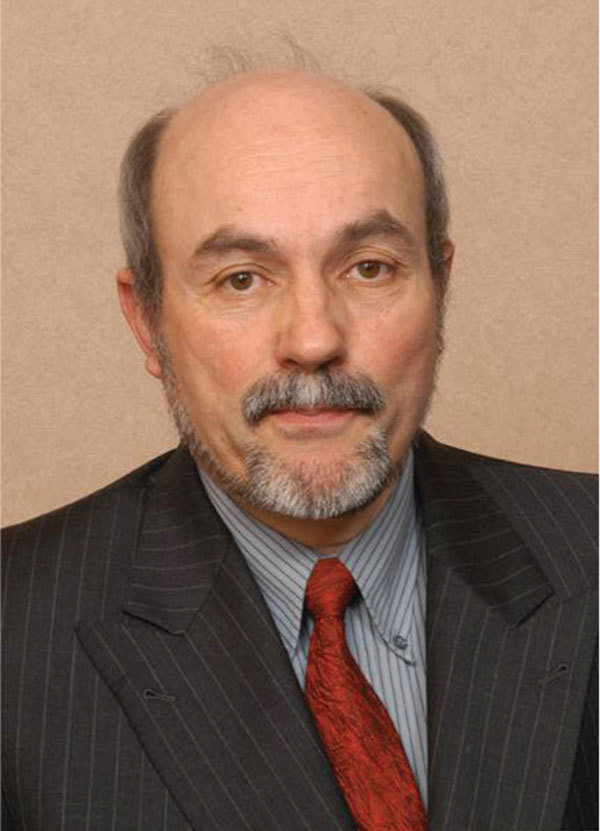
*Johannes Ring (Executive Editor)*

**Lanny Rosenwasser:**
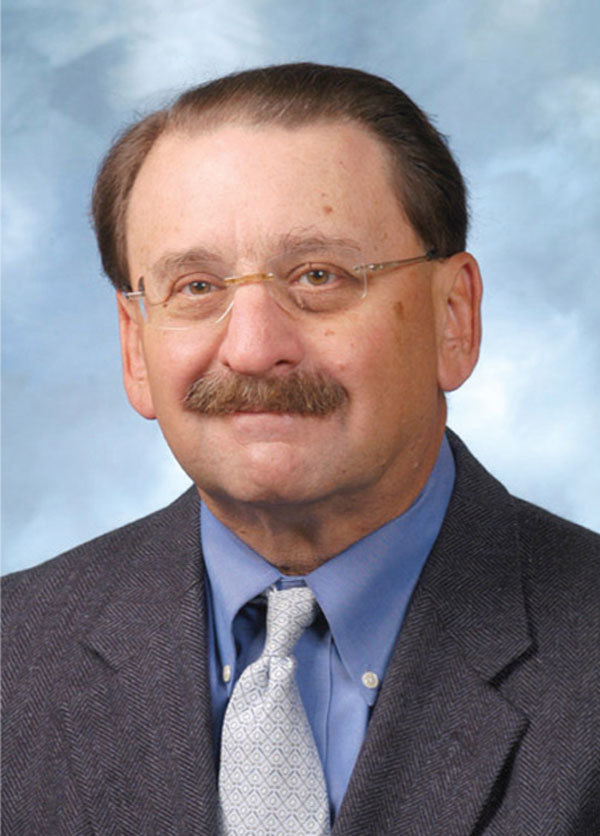
*Lanny Rosenwasser (Editor-in-Chief)*

As was noted in last month's editorial, significant progress has been made in the past year in many aspects of Allergy and Immunology worldwide, especially in areas that are spearheaded by WAO.

One of the critical aspects of our discipline is the use of allergen specific immunotherapy as treatment for allergic diseases. Originally, the empiric institution of this therapy was found to have profound influences on diseases such as allergic catarrh or hay fever. The current state of the art has been reviewed in many venues, and significant information over the past 20 years has accumulated that a particular route of administering immunotherapy, namely sublingual immunotherapy (SLIT), has a particularly strong profile in terms of safety without sacrificing the usual efficacy associated with standard injection subcutaneous immunotherapy (SCIT). Potential mechanisms to underline this approach to specific immunotherapy include the route of mucosal administration as being a particularly effective form of inducing immune tolerance without some of the potential risks of parenteral administration. The allergy diagnosis, the indication, initiation, and follow-up observation of SLIT is the absolute domain of an experienced allergist.

The WAO has taken a significant interest in potential mechanisms and efficacy surrounding SLIT immunotherapy. A number of important initiatives have been undertaken by WAO to examine the proper mode of study for efficacy and safety within the burgeoning number of worldwide studies concerning SLIT. The dosage, clinical efficacy, safety and practical implications of this form of therapy are profound since the eventual administration of SLIT has significant advantages over standard SCIT, and the potential use of this approach worldwide becomes an issue of great interest for pharmacoeconomic and practical reasons.

Over the past year, significant evaluation and speculation concerning this form of therapy has appeared in many venues and journals, including our own journal where SLIT was reviewed and pollen specific SLIT was evaluated in an observational study of the oral allergy syndrome. Based on the initiatives and interest of the WAO, it is anticipated that a number of position statements and summaries concerning the current state of the art of SLIT and standards utilized for evaluation of SLIT trials that are underway will be forthcoming this year.

The development of proper dosages and specific indications for respiratory and other allergic diseases treatment by SLIT will be critical. Acceptance of this kind of therapy in areas where IT has not made significant inroads and has not been approved, beyond Europe, will also be of critical importance. The use of this therapy in North America, Latin America, the Middle East, Africa and Asia, will be an interesting development, which will be followed closely for the next few years. It is anticipated that a number of papers and findings concerning SLIT will be published in the next year within our WAO Journal, and we will keep abreast of developments published in other areas of allergy and immunology on this important subject. We anticipate and welcome submission of original and mechanistic studies concerning SLIT to this Journal.

Johannes Ring

(Executive Editor)

Lanny Rosenwasser

(Editor-in-Chief)

